# Supplementary material for: IceR improves proteome coverage and data completeness in global and single-cell proteomics
Source: Nat Commun. 2021 Aug 9;12:4787. doi: 10.1038/s41467-021-25077-6 (PMC8352929; doi:10.1038/s41467-021-25077-6)
Supplement: Supplementary file 1 — Supplementary Information [file 41467_2021_25077_MOESM1_ESM.pdf]

Supplementary Information

IceR improves proteome coverage and data completeness in global and single-cell proteomics

Mathias Kalxdorf, Torsten Müller, Oliver Stegle and Jeroen Krijgsveld

Supplementary Note 1

IceR analysis pipeline

Below, we explain the consecutive steps performed by IceR in detail, accompanying Suppl Fig 1 showing these steps graphically. In addition to describing the analysis workflow, performance of IceR is illustrated based on its processing of a published spike-in data set (iPRG 2015[1]). In that study, yeast digests were spiked (n=3) with different concentrations of six exogenous marker proteins. Its limited complexity typically allows good performances of analysis workflows to detect spiked proteins. As this data set was previously used to evaluate DeMix-Q[2], it enabled us to directly compare results of DeMix-Q and IceR. MaxQuant results of the same data were used as a reference.

To launch an IceR analysis, user input is only needed to define minimal m/z and retention time windows, minimal kernel density resolution, and number of threads to be used (Suppl fig 12). Default values can be used for most analyses. Next, IceR automatically proceeds through the following 13 steps, aligning with the panel numbers in Suppl. Fig 1:

1.) Estimation of feature alignment windows. In the iPRG2015 data set, peptide sequences could be assigned to only 20% of all features detected by MaxQuant, thereby leaving 80% of features unused (Supplementary Fig. 3a). IceR estimated suitable RT- and m/z-feature alignment windows by determining deviations in RT and m/z for identified peptide features over samples. Here, the m/z- and RT-alignment windows were determined to be at least 0.35 mDa and 0.35 min, respectively (Supplementary Fig. 3b).
2.) MaxQuant features with same peptide sequence, charge-state, and PTM over samples are aggregated into a new IceR feature with median RT and m/z. Peptide features from samples with m/z or RT deviating from these medians by more than the defined alignment windows are excluded.
3.) For an increased statistical power and quantification accuracy, for every IceR feature an expected +1-isotope feature is added. Furthermore, for estimation of background noise per quantification, a decoy feature with an arbitrarily shifted extraction window is generated for every IceR target feature.
4.) The original location (RT and m/z and if available IM) for every IceR feature in every sample is determined, and its distance to the aggregate IceR feature locations is stored as a correction factor. If MaxQuant feature information is lacking, this location has to be estimated. For that purpose, m/z- and RT-corrections are modelled by sample-specific random forests (RF) and generalized additive models (GAM), respectively (Supplementary Fig. 3c,d). The majority of iPRG2015 samples show consistent chromatographic elution profiles while e.g. sample 7 shows an increasing shift in retention times which would prevent PIP.
5.) Unsequenced MaxQuant features that fall into the alignment windows of an IceR feature are regarded as missed peptide features. For these, sequence information is transferred based on feature-based PIP. In case of the iPRG2015 data set, sequence information could be transferred by feature-base PIP for about 34% of IceR features lacking an identity in the iPRG2015 data set (Supplementary Figure 2a). For all remaining IceR features per sample (on average 13%) that even lack detection of any MaxQuant feature, sequence information could be transferred by ion-based PIP.
6.) Decoy features are used to model and estimate quantification background noise by counting and summing up intensities of ions that randomly fall into DICE-windows (Supplementary Fig. 3e).
7.) Locations of peaks (accumulations of ions) around the expected DICE-windows, based on the first global alignment of IceR features, are detected by kernel density estimations. Kernel density plots and detected 2D peaks for a peptide (LWSAEIPNLYR) of the spiked protein lacZ are shown in Supplementary Fig. 2b. MaxQuant (match-between-runs enabled) detected this peptide only in samples 4 – 9 while IceR was able to detect the correct peak in all 12 samples after its 2-step alignment procedure (Supplementary Fig. 2b).

8.) IceR tries to select a peak for every aggregated feature in all samples. In case of IM data, available ions are prefiltered requiring their IM to fall within the expected IM window. For samples with detected MaxQuant feature, the peak closest to the expected feature location is selected as long as it is formed by n ions (n = median of observed decoy ion counts) and is located within the expected DICE-window. These peaks are classified as ‘known’. In all other cases, IceR picks the peak closest to all known locations in other samples as long as it is not overlapping with any other peak in these samples, its m/z is not deviating more than 3 times the m/z alignment window and its RT is not deviating more than the RT alignment window (Supplementary Fig. 2b). If no peak fulfils these criteria for a sample, the expected DICE-window for this IceR feature is used. The alignment approach of IceR reduced the median deviations of RT and m/z between samples by more than 3-fold and 2-fold, respectively (Supplementary Fig. 2c). After peak selection, all ions within the selected DICE-windows are counted and their intensities are summed.

9.) The significance of ion accumulation per quantification is determined by comparing the number of observed ions in DICE-windows against background noise ion count distributions. Resulting quantification pvalues thus indicate if more ions than expected by chance accumulated within respective DICE-windows. Features with significant accumulations (pvalue<0.05) can be regarded as truly present. The quality of each quantification is further evaluated based on signal to noise ratios. For the current data set, almost all quantifications show significant ion accumulation (median $-\log_{10}$ pvalues of 5, Supplementary Fig. 3f) and a generally good signal to noise ratio (median S/N ~ 14, Supplementary Fig. 3g). +1-isotope IceR features that show no significant accumulation of ions (pvalue>0.05) in any sample are removed.

10.) Peak selections are verified and outliers removed. Two filters are applied: 1.) Features showing significantly (pvalue<0.05) increased interquartile ranges for peak RT or peak m/z between samples are completely removed. These numbers are usually below 1% (Supplementary Fig. 3h) 2.) Features showing significantly (pvalue<0.05) deviating peak RT or m/z in individual samples are excluded. An additional filter is applied for +1-isotope IceR features by detecting outliers that show a significant (pvalue<0.05) deviation of peak RT or m/z between the monoisotopic and +1-isotope IceR features (typically below 20% (Supplementary Fig. 3i)).

11.) For every IceR analysis, accuracy of peak selection is estimated by performing a false discovery rate (FDR) analysis. Therefore, 500 IceR features with peaks classified as ‘known’ are randomly selected per sample, their true peak locations are masked (treated as if no MaxQuant feature was detected) and it is then evaluated, how often the algorithm ends up selecting a wrong peak with deviating intensity. Typically, peak selection FDR is below 1%. In case of the iPRG2015 data set, an average FDR of 0.6% was estimated (Supplementary Fig. 2d).

12.) Optionally, the background noise models can be used to impute missing feature quantifications. Missing values that resulted from the applied filtering criteria are not imputed because of likely wrong peak selection. In the iPRG2015 data set, on average 98% of monoisotopic peptide features could be directly quantified by DICE and remaining 2% of quantifications were imputed (Supplementary Figure 2e). In contrast, MaxQuant could estimate abundances only for 86% of all identified peptides. Additionally added +1-isotope features by IceR could be directly quantified by DICE in 84% of cases while 2% of quantifications were imputed. The remaining 14% of +1-isotope IceR features are left with missing values due to exclusion by filtering criteria.

13.) Finally, protein-level quantification is obtained by aggregating feature level quantifications. By default, aggregation is performed by Top-3[3], total sum intensity and MaxLFQ[4] approaches.

Following IceR requantification, we compared the results of our workflow with the outputs generated by MaxQuant (match-between-runs enabled) as well as with published DeMix-Q results. Numbers of identified proteins and peptides were comparable between all three approaches (Supplementary Fig. 3j,k). However, MaxQuant resulted in 12-fold more missing values compared to DeMix-Q and IceR (Supplementary Fig. 2f,g). DeMix-Q showed lowest coefficients of variation (CVs) of peptide

quantifications, however, IceR resulted in almost 2-fold more available quantification events including more variable low abundant features (Supplementary Fig. 3l). When focusing on feature quantifications with significant ion accumulation and signal-to-background ratio (quantification pvalue < 0.01, S/B > 4-fold), CVs in IceR were comparable to DeMix-Q results. To take advantage of the highly increased numbers of peptide quantifications available in DeMix-Q and IceR results, differential expression (DE) analyses were performed on peptide-level using peptide-level expression-change averaging (PECA[5]) for all tools. Additionally, as peptide-level DE can result in reduced sensitivity in case of high data sparsity as typically returned from standard label-free quantification workflows, DE analyses were also performed on protein-level for MaxQuant and IceR. The limited complexity of the data set enabled similar performances for detecting true positives by all three tools (Supplementary Fig. 3m,n). However, IceR resulted in most accurate and precise protein abundance ratio estimations (Supplementary Fig. 2h) and enhanced statistical power for DE analyses compared to DeMix-Q (Supplementary Fig. 2i).

Supplementary Figures

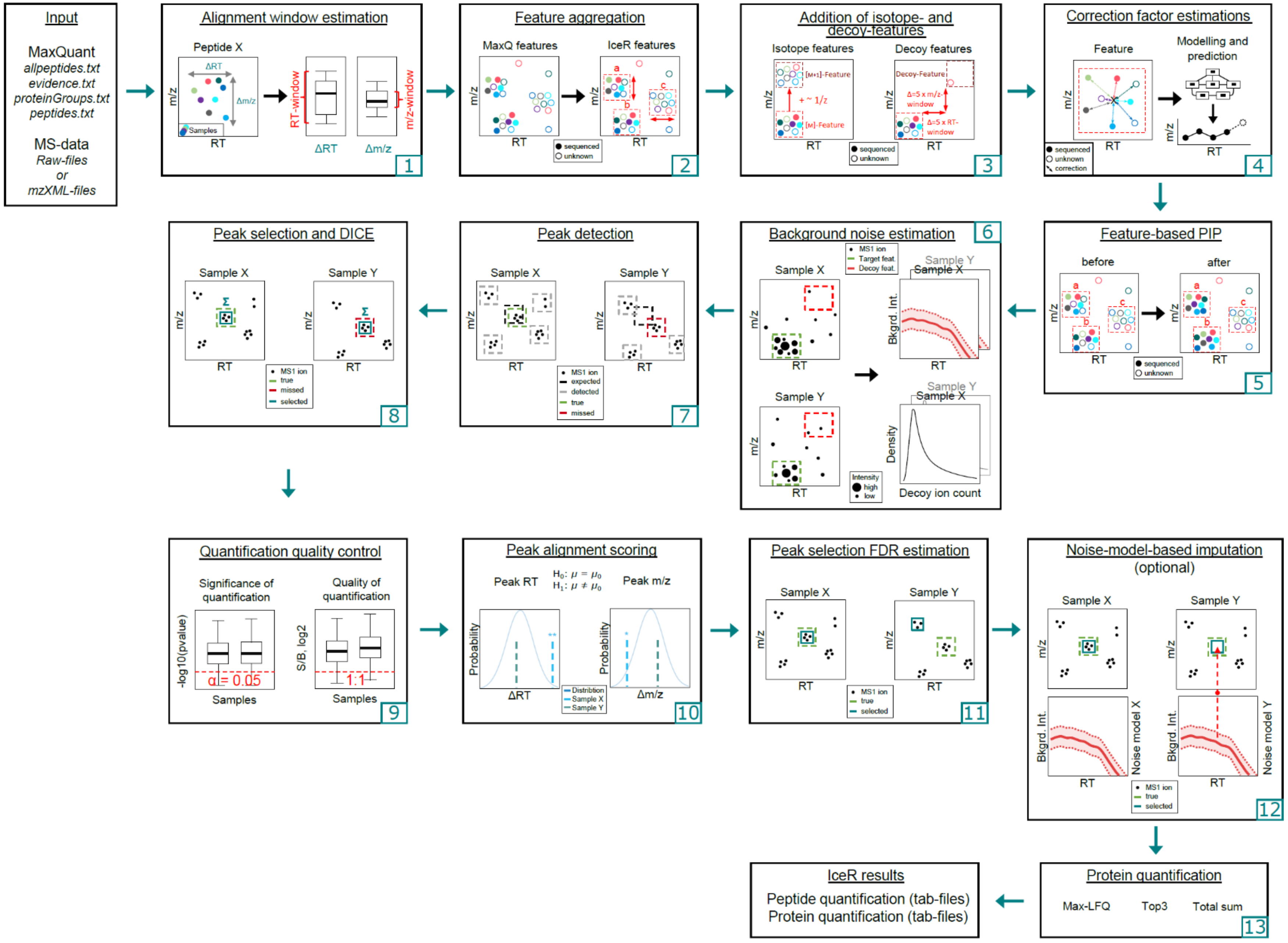

Supplementary Fig. 1 – IceR workflow in detail.

IceR starts from MaxQuant result files and MS-data in the mzXML format. If MS-data is supplied as Thermo Raw-files, they can be automatically converted to mzXML format by MSConvert from ProteoWizard. The IceR workflow consists of 13 steps, summarized here and described in more detail in Supplementary Note 1.

Step 1) Alignment windows are determined by estimating deviations of observed retention time (RT) and calibrated m/z for peptides identified in samples.

Step 2) IceR features are defined by aggregating MaxQuant features across samples using defined alignment windows.

Step 3) Introduce decoy and +1-isotope features per IceR feature.

Step 4) RT- and m/z-correction factors are extracted per IceR feature and sample. If a feature was not detected by MaxQuant for an IceR feature in an individual sample, data modelling approaches are used to predict correction factors.

Step 5) Peptide sequence information within IceR features is propagated from sequenced MaxQ features between samples.

Step 6) Background noise, which is expected per IceR feature quantification, is estimated by counting and summing up intensities of ions that fall into decoy feature DICE-windows.

Step 7) Normal kernel density estimation to detect accumulations of ions (peaks) in RT- and m/z-space around the expected DICE-window per IceR feature and sample.

Step 8) Peaks are selected by applying a robust selection algorithm. Ions falling into selected DICE-windows are counted, distinguished into signal- or background-ions and corresponding intensities are summed.

Step 9) The significance of ion accumulation per quantification is determined by comparing the number of observed ions in DICE-windows against expected background noise ion count distributions (i.e. observed decoy feature ions). The quality of each quantification is further evaluated based on signal to noise ratios.

Step 10) Peak selection over samples is statistically evaluated and significant outliers are excluded.

Step 11) A peak selection false discovery rate (FDR) is estimated per sample.

Step 12) Optional: Missing quantifications of IceR features can be imputed using the decoy feature-based sample-specific background noise models.

Step 13) Peptide quantifications are aggregated to protein quantifications using the Top3, total sum intensity and MaxLFQ approach.

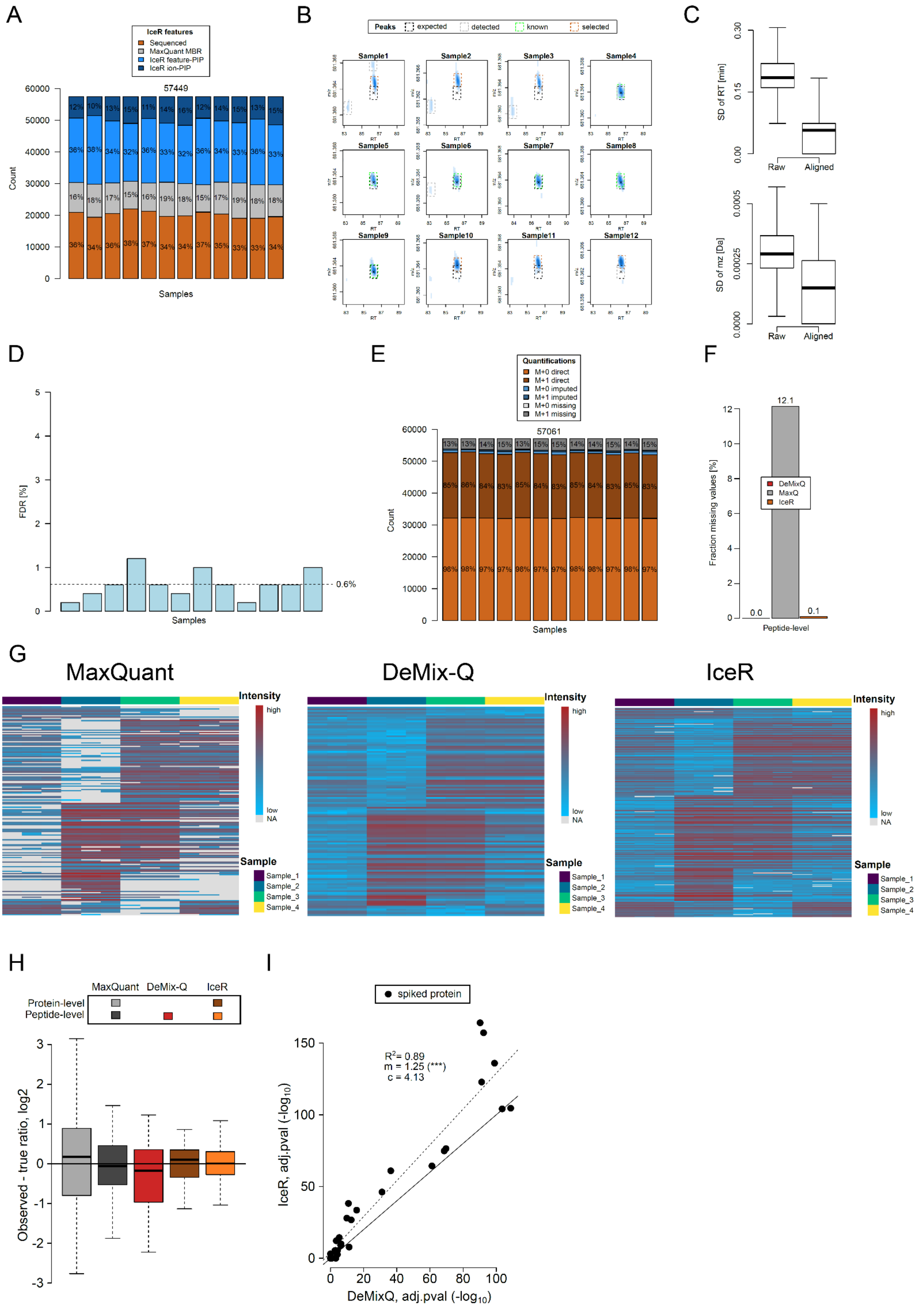

Supplementary Fig. 2 – IceR quality control assessed by processing of iPRG 2015 data.

a, Numbers of IceR features per sample identified by MSMS, by the match-between-runs algorithm of MaxQuant, by feature-based PIP or ion-based PIP by IceR. **b**, Density plot per sample visualizing accumulation of peptide ions (LWSAEIPNLYR) of the spiked protein lacZ detected by normal kernel density estimation. Black dashed boxes indicate expected 2D peak locations per sample. Detected 2D peaks are indicated with grey dashed boxes. True known peaks (by MS/MS identifications or PIP) in respective samples are indicated in green. These peaks are also selected by the peak selection algorithm of IceR for the respective sample. Peaks that were selected by the peak selection algorithm of IceR in samples lacking knowledge of the true peak location are indicate in orange. **c**, Deviation of IceR feature m/z and RT over n=12 samples before (raw) and after (aligned) alignment. Center line, median; box limits, upper and lower quartiles; whiskers, 1.5x interquartile range. **d**, False peak discovery rate estimation by masking location of 500 randomly picked IceR features with true identification per sample and evaluating how often a wrong peak is selected. Dashed line indicates mean false discovery rate (FDR). **e**, Numbers of monoisotopic (M+0, light colour) and +1-isotopic (M+1, dark colour) IceR features per sample quantified by DICE (orange), quantified by background noise model-based imputation (blue) or with missing values (grey). **f**, Fraction of missing values on peptide-level in DeMix-Q (red), MaxQuant (grey) and IceR (orange) outputs. **g**, Heatmap representation of quantified peptides of six spiked proteins in four tool samples (n=3) in MaxQuant (left), DeMix-Q (middle) and IceR (right) results. Peptides are ordered over all three outputs. Low abundance peptides are coloured blue, high abundant peptides are coloured red, and missing values are indicated in grey. **h**, Boxplot representation of $\log_2$ deviations between estimated abundance ratios and true spike-in ratios of respective pairwise differential expression analyses for the n=6 spiked proteins. Center line, median; box limits, upper and lower quartiles; whiskers, 1.5x interquartile range. **i**, Comparison of adjusted pvalues for spiked proteins in pairwise differential expression analyses in DeMix-Q and IceR outputs. Coefficient of determination (R^2), slope and x-intercept of the linear model fit are indicated. Significance for the deviation of the slope from the diagonal determined by linear regression. *** indicates pvalue < 0.001.

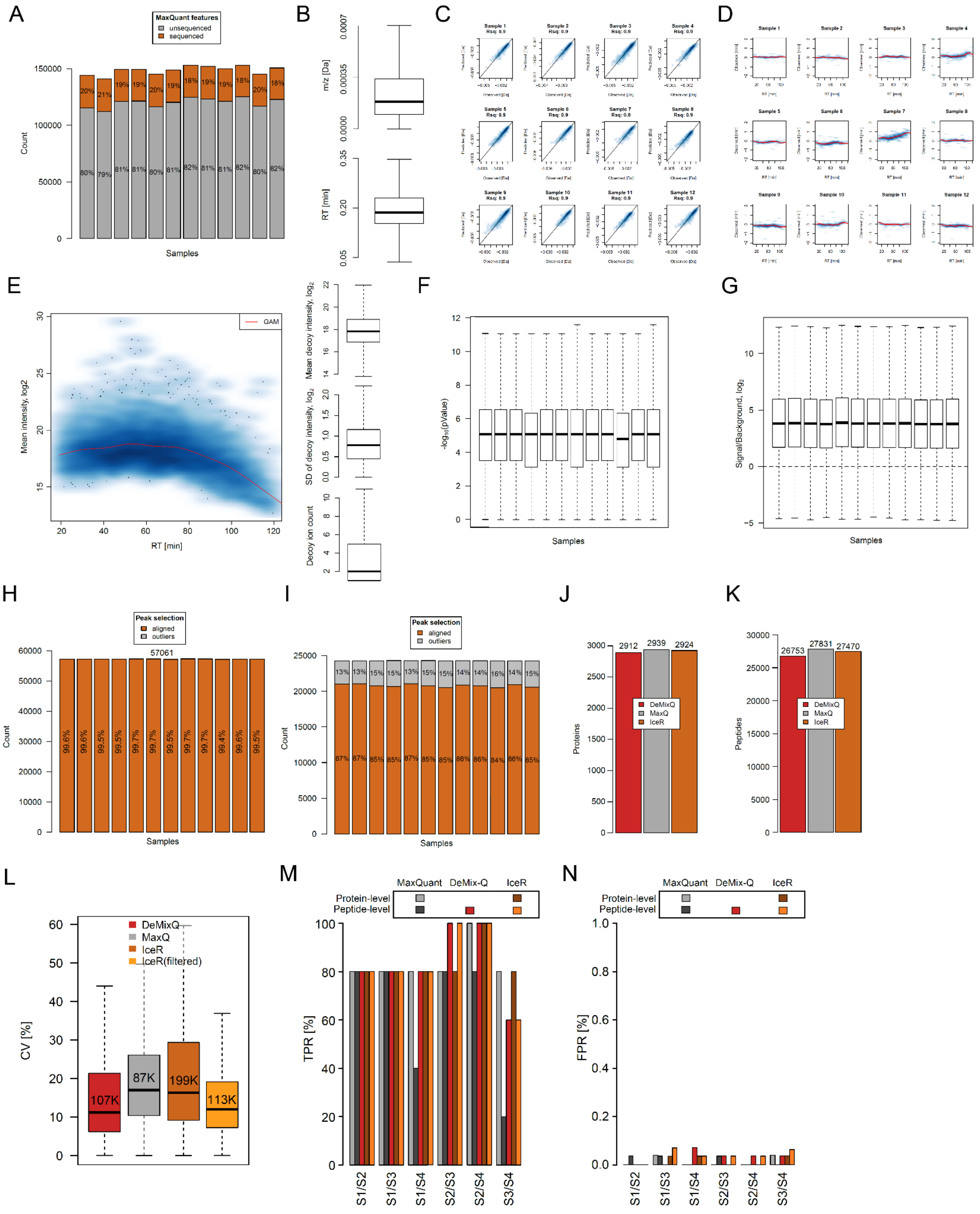

Supplementary Fig. 3 – Detailed IceR quality control assessment for iPRG 2015 data.

a, Numbers of detected features with and without peptide sequence by MaxQuant per sample. **b**, Deviations in m/z and retention time between samples used to automatically define required feature alignment windows for IceR workflow (75%-quantile of m/z deviations and upper whisker of RT deviations) based on n=33717 peptide features. Center line, median; box limits, upper and lower quartiles; whiskers, 1.5x interquartile range. **c**, Smoothed scatterplot visualizing 20 % of observed feature m/z corrections over sample-specific random forest model predictions. Coefficient of determination (Rsq) is indicated. **d**, Smoothed scatterplot visualizing sample- and chromatographic retention time-specific generalized additive models (GAM) fitted to observed RT corrections per feature and sample. **e**, Smoothed scatterplot visualizing observed mean ion intensities within decoy ion direct ion current extraction windows over chromatographic retention time. Fitted GAM is indicate by a red line. Boxplot representations of mean ion intensities of decoy features, standard deviation of ion intensities of decoy features and numbers of ions per decoy feature are plotted (for n=33717 decoy features). Center line, median; box limits, upper and lower quartiles; whiskers, 1.5x interquartile range. **f**, Boxplot visualization of determined significances of ion accumulations of n=57061 IceR feature quantifications per sample. Center line, median; box limits, upper and lower quartiles; whiskers, 1.5x interquartile range. **g**, Boxplot visualization of signal to background ratios (log_2) of n=57061 IceR feature quantifications per sample. **h**, Absolute and relative fraction of IceR features per sample for which selected peaks are aligned to (orange) or are significantly deviating from (grey) corresponding peaks in all other samples. **i**, Absolute and relative fraction of +1-isotope IceR features for which peaks were selected with significant RT and/or m/z deviation compared to its respective monoisotopic feature. **j**, Number of proteins identified with at least 2 features by DeMix-Q (red), MaxQuant (grey) and IceR (orange). **k**, Number of peptides identified by DeMix-Q (red), MaxQuant (grey) and IceR (orange). **l**, Boxplot of coefficients of variation (CV) of peptide quantifications in DeMix-Q (red), MaxQuant (grey), IceR (orange), and filtered IceR (pvalue of ion accumulation < 0.01, signal to background >= 4, light orange) results. Numbers of available CVs per boxplot are indicated. Center line, median; box limits, upper and lower quartiles; whiskers, 1.5x interquartile range. **m**, True positive rates (TPR %) of pairwise differential expression analyses on protein- and peptide-level in MaxQuant (grey), DeMix-Q (red) and IceR (orange) data. **n**, As in m but showing corresponding false positive rates (FPR %)

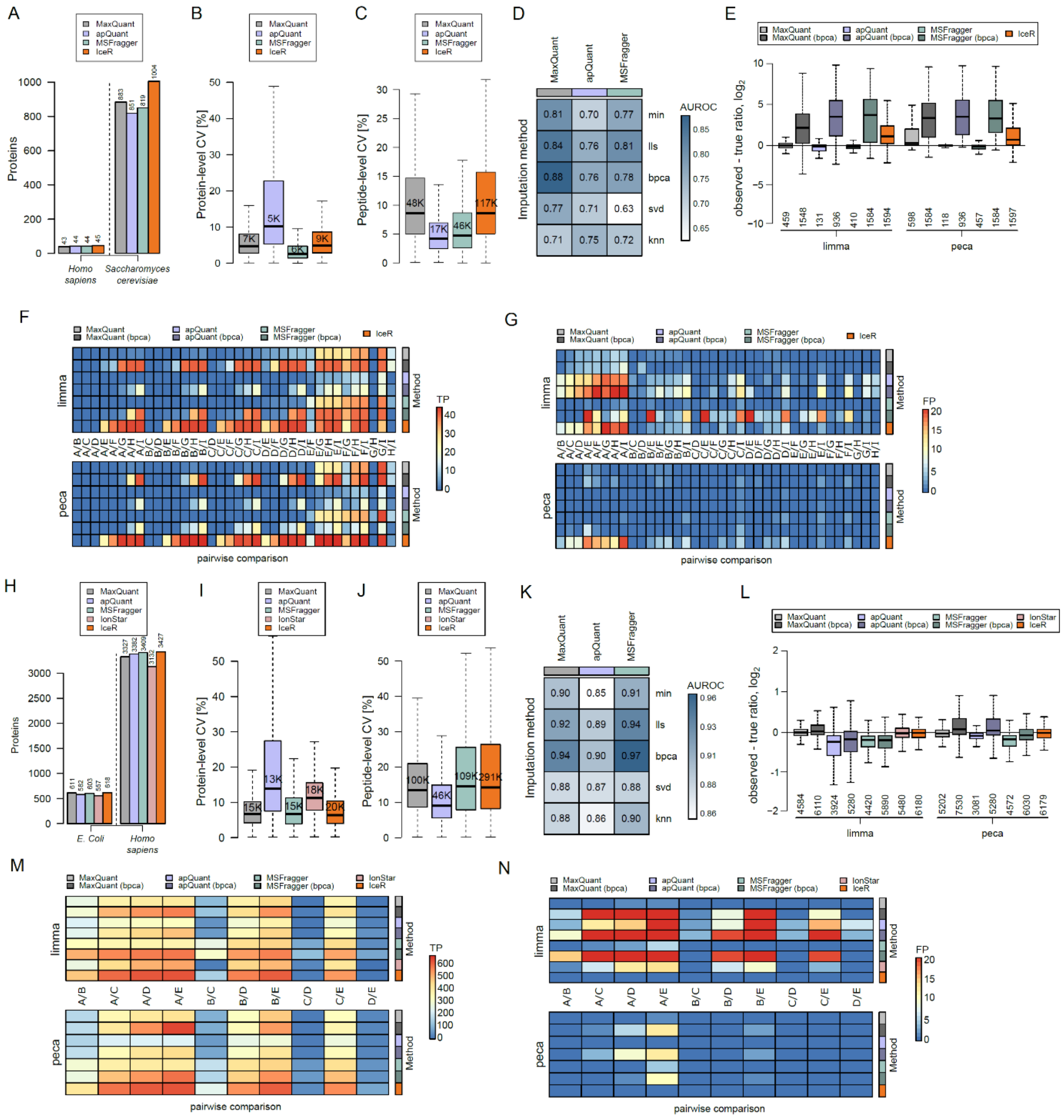

Supplementary Fig. 4 – Evaluation of IceR based on two publicly available tool data sets.

Supplementary analysis results for the tool data set of Ramus et al. (a-g) and Shen et al. (h-n). **a**, Numbers of quantified proteins in MaxQuant (grey), apQuant (purple), MSFragger (green), and IceR (orange) results. **b**, Coefficient of variation of protein quantifications in MaxQuant (grey), apQuant (purple), MSFragger (green), and IceR (orange) results. Numbers of available CVs per boxplot are indicated. Center line, median; box limits, upper and lower quartiles; whiskers, 1.5x interquartile range. **c**, as in b but showing CVs of peptide quantifications. **d**, Heatmap representation of areas under the ROC (AUROC) for all (36) pairwise differential expression analyses using limma after missing value imputation by five commonly used imputation methods for MaxQuant (grey), apQuant (purple), and MSFragger (green) data. Imputation by bpca resulted in overall best AUROC. **e**, Deviation of determined from true protein abundance ratios of pairwise differential expression analyses on protein-level (limma) and peptide-level (peca) in MaxQuant (grey), MaxQuant with bpca imputation (dark grey), apQuant (purple), apQuant with bpca imputation (dark purple), MSFragger (green), MSFragger with bpca imputation (dark green), and IceR (orange) data. Numbers of spiked proteins for which abundance ratios could be determined per condition are indicated. Center line, median; box limits, upper and lower quartiles; whiskers, 1.5x interquartile range. **f**, Numbers of detected true positives on protein-

level (limma) and peptide-level (peca) by respective quantification method for each individual pairwise comparison. Spike-in amounts in fmol/µL: A = 0.05, B = 0.125, C = 0.25, D = 0.5, E = 2.5, F = 5, G = 12.5, H = 25, I = 50. **g**, As in f but showing numbers of detected false positives. **h**, Numbers of quantified proteins in MaxQuant (grey), apQuant (purple), MSFragger (green), IonStar (pink), and IceR (orange) results. **i**, Coefficient of variation of protein quantifications in MaxQuant (grey), apQuant (purple), MSFragger (green), IonStar (pink), and IceR (orange) results. Numbers of available CVs per boxplot are indicated. Center line, median; box limits, upper and lower quartiles; whiskers, 1.5x interquartile range. **j**, as in i but showing CVs of peptide quantifications. **k**, Heatmap representation of areas under the ROC (AUROC) for all (10) pairwise differential expression analyses using limma after missing value imputation by five commonly used imputation methods for MaxQuant (grey), apQuant (purple), and MSFragger (green) data. Imputation by bpca resulted in overall best AUROC. **l**, Deviation of determined from true protein abundance ratios of pairwise differential expression analyses on protein-level (limma) and peptide-level (peca) in MaxQuant (grey), MaxQuant with bpca imputation (dark grey), apQuant (purple), apQuant with bpca imputation (dark purple), MSFragger (green), MSFragger with bpca imputation (dark green), IonStar (pink), and IceR (orange) data. Numbers of spiked proteins for which abundance ratios could be determined per condition are indicated. Center line, median; box limits, upper and lower quartiles; whiskers, 1.5x interquartile range. **m**, Numbers of detected true positives on protein-level (limma) and peptide-level (peca) by respective quantification method for each individual pairwise comparison. Spike-in amounts relative to human backround in %: A = 3, B = 4.5, C = 6, D = 7.5, E = 9. **n**, As in m but showing numbers of detected false positives.

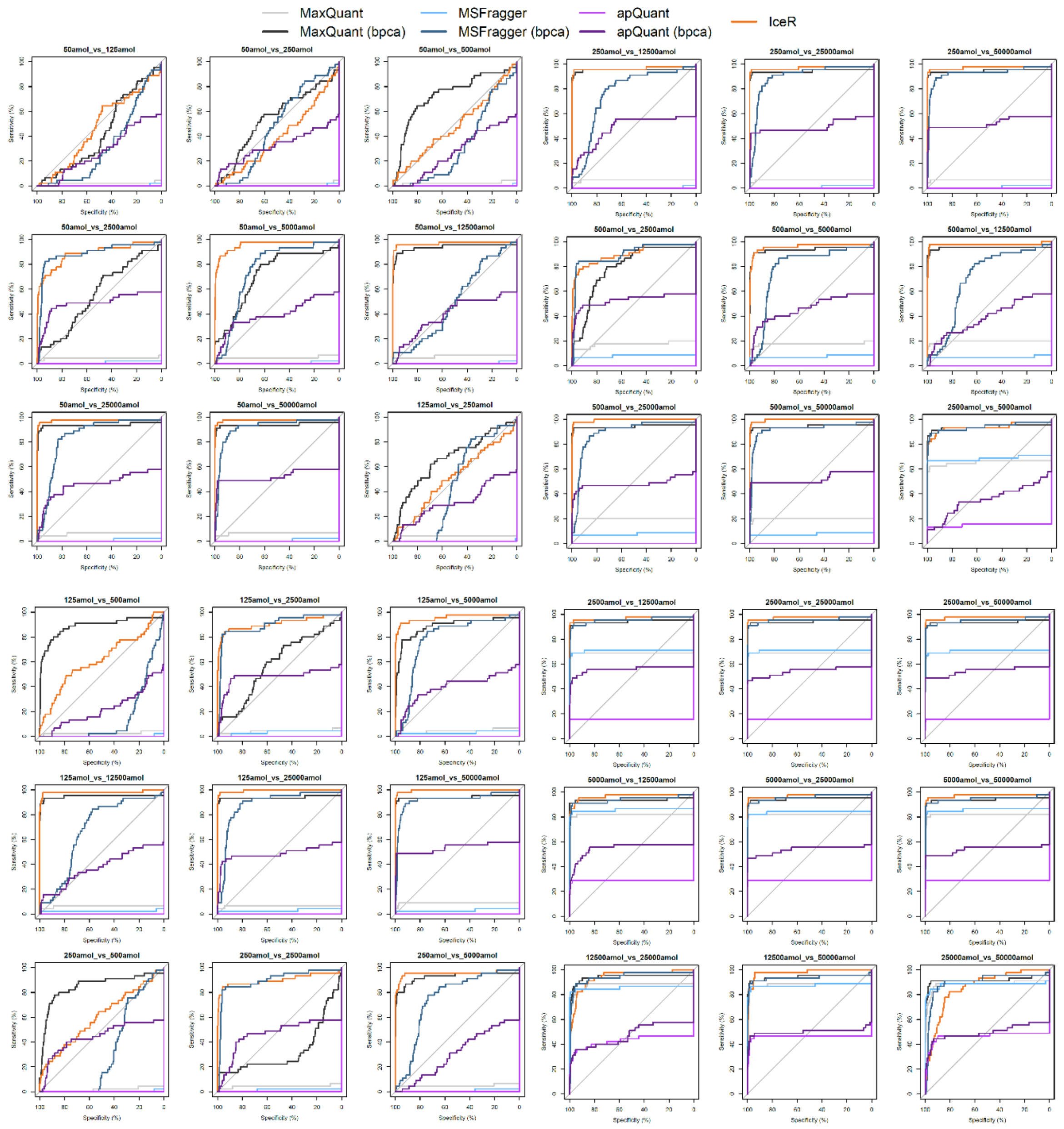

Supplementary Fig. 5 – All pairwise ROC curves for data set of Ramus et al..

All pairwise ROC curves on protein-level differential testing for MaxQuant (grey), MaxQuant with bpca imputation (dark grey), apQuant (purple), apQuant with bpca imputation (dark purple), MSFragger (blue), MSFragger with bpca imputation (dark blue), and IceR (orange) data.

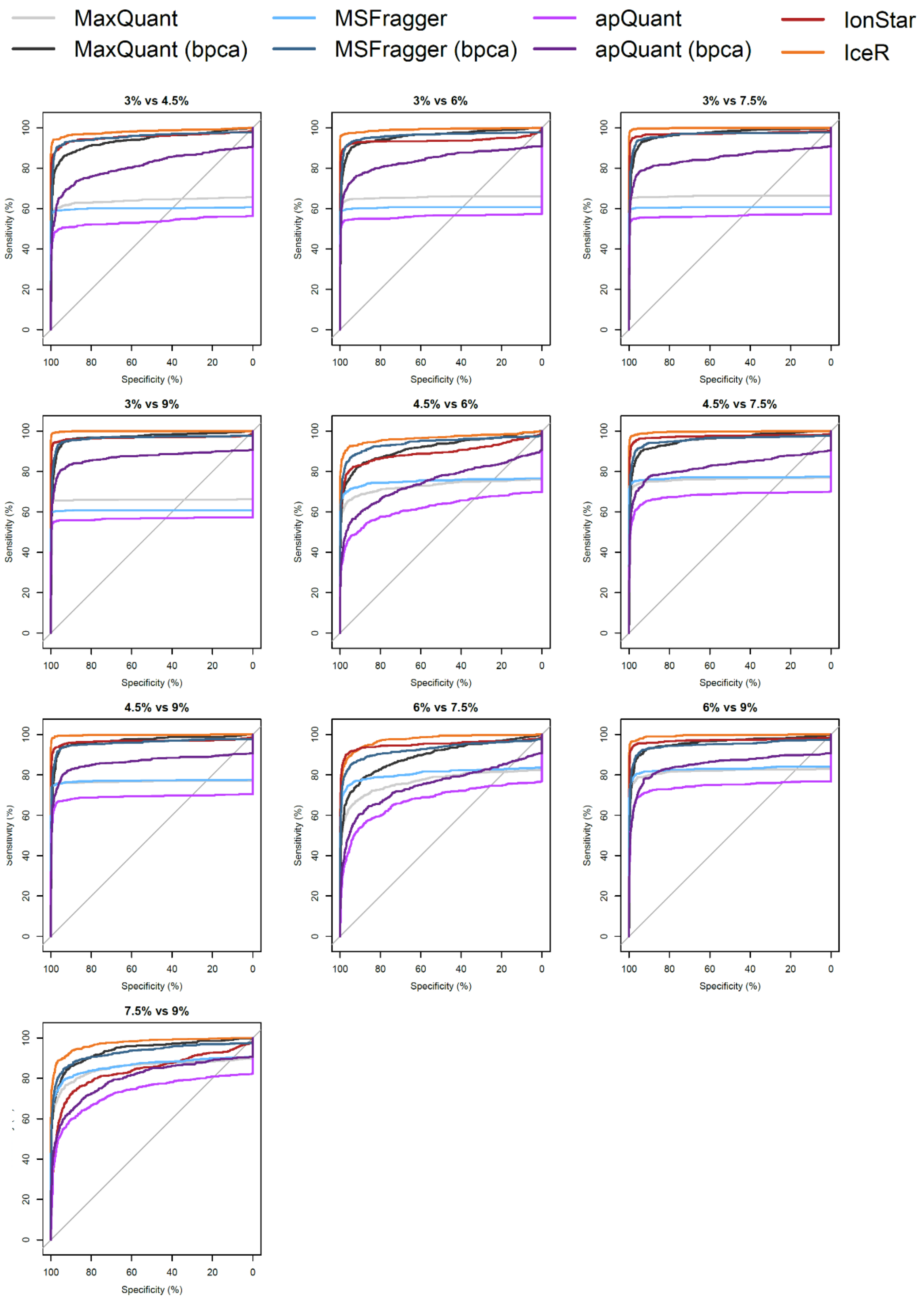

Supplementary Fig. 6 – All pairwise ROC curves for data set of Shen et al..

All pairwise ROC curves on protein-level differential testing for MaxQuant (grey), MaxQuant with bpca imputation (dark grey), apQuant (purple), apQuant with bpca imputation (dark purple), MSFragger (blue), MSFragger with bpca imputation (dark blue), IonStar (dark red), and IceR (orange) data.

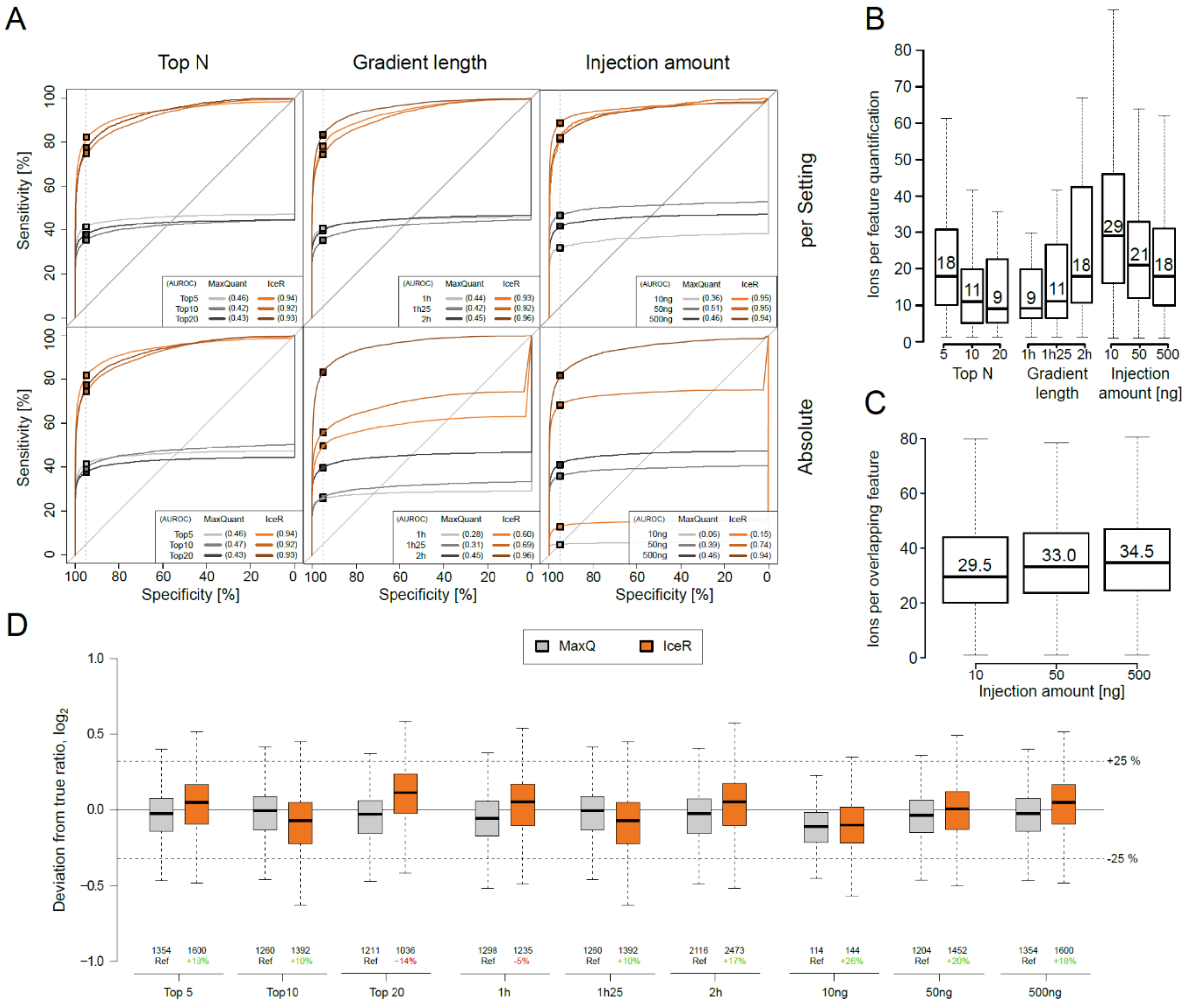

Supplementary Fig. 7 – IceR enables robustly improved sensitivity for a wide range of typically varied MS analysis parameters.

a, ROC curves for pairwise differential expression analyses in MaxQuant (grey) and IceR (orange) data at respective Top N (left, Top5 to Top20), gradient length (center, 1h to 2h) and sample injection amount (right, 10 ng to 500 ng). Upper panels show ROC curves with number of expected true positives set to the number of identified spiked proteins in MaxQuant data per setting. Lower panels show ROC curves with number of expected true positives set to the absolute maximum number of identified spiked proteins per experiment. Respective areas under the ROC (AUROC) are indicated. Respective sensitivities at 95 % specificity are indicated by squares. **b**, Boxplot representation of available data points per feature quantification in respective experiment and setting. Median counts are indicated. Center line, median; box limits, upper and lower quartiles; whiskers, 1.5x interquartile range. **c**, As in **b** but showing numbers of available data points per quantification of features detected over all sample injection amounts. **d**, Deviation of observed from true protein abundance ratio in pairwise differential expression analyses in MaxQuant (grey) and IceR (orange) outputs in respective experiment and setting. Absolute numbers and relative fractions compared to MaxQuant results of spiked proteins for which abundance ratios could be determined per condition are indicated. Center line, median; box limits, upper and lower quartiles; whiskers, 1.5x interquartile range.

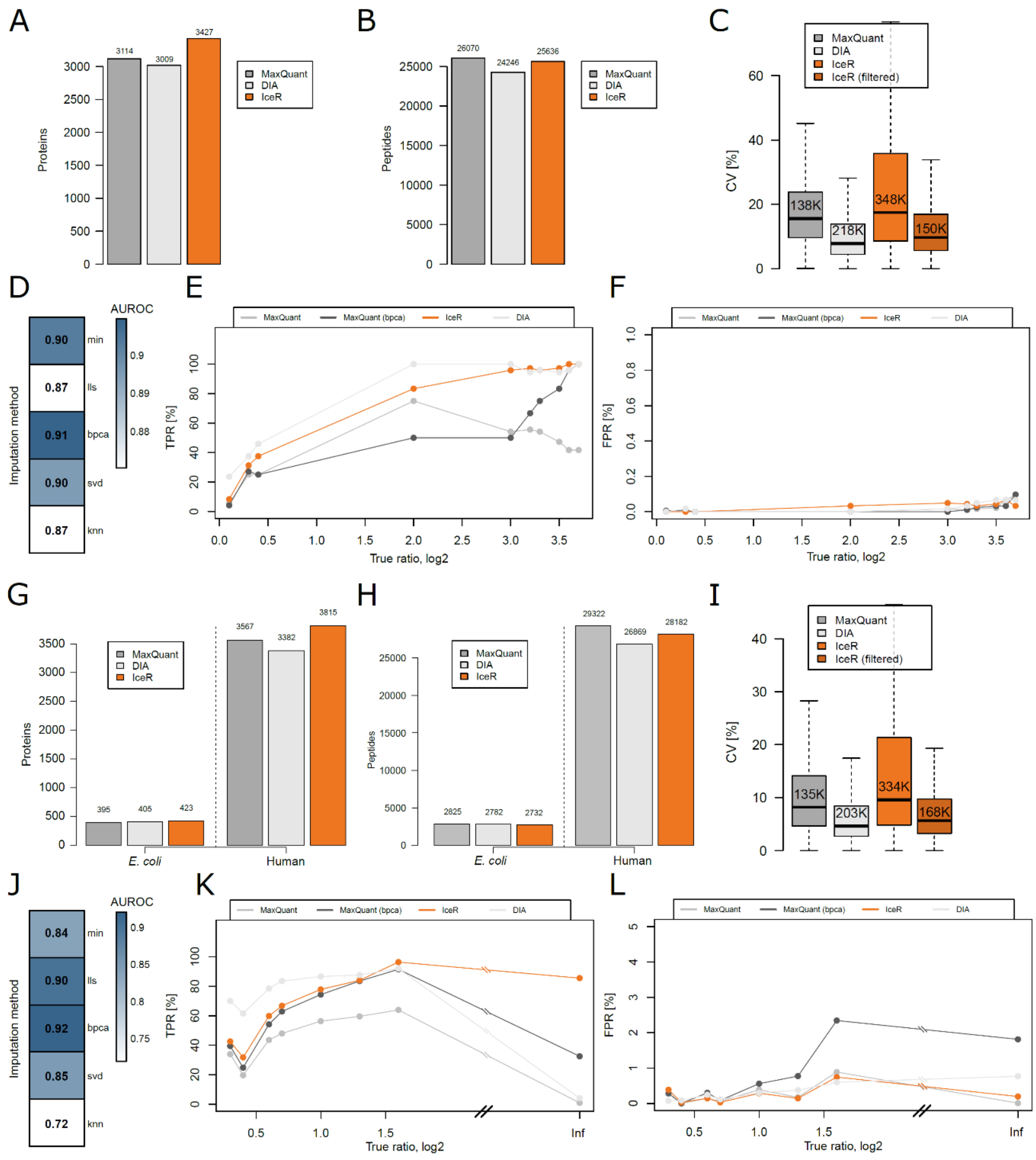

Supplementary Fig. 8 – Comparing performance of IceR and DIA for label-free proteomics.

Supplementary analysis results for the tool data set of Bruderer et al. (a-e) and an in-house generated tool data set (f-j). **a**, Number of quantified proteins in MaxQuant (grey), DIA (red) and IceR (orange) data. **b**, Number of quantified peptides in MaxQuant (grey), DIA (red) and IceR (orange) data. **c**, Coefficient of variation of peptide quantifications in MaxQuant (grey), DIA (red), IceR (orange) and filtered IceR (pvalue of ion accumulation < 0.05, signal to background >= 4, dark orange) results. Numbers of available CVs per boxplot are indicated. Center line, median; box limits, upper and lower quartiles; whiskers, 1.5x interquartile range. **d**, Heatmap representation of areas under the ROC (AUROC) for all (28) pairwise differential expression analyses using limma after missing value imputation by five commonly used imputation methods for MaxQuant data. Imputation by bpca resulted in best AUROC. **e**, Mean true positive rates for MaxQuant (grey, protein-level DE), DIA (red, peptide-level DE) and IceR (orange, peptide-level DE) outputs over true spike ratio of pairwise differential expression analyses. **f**, Mean false positive rates for MaxQuant (grey, protein-level DE), DIA (red,

peptide-level DE) and IceR (orange, peptide-level DE) outputs over true spike ratio of pairwise differential expression analyses. **g**, Number of quantified proteins in MaxQuant (grey), DIA (red) and IceR (orange) data. **h**, Number of quantified peptides in MaxQuant (grey), DIA (red) and IceR (orange) data. **i**, Coefficient of variation of peptide quantifications in MaxQuant (grey), DIA (red), IceR (orange) and filtered IceR (pvalue of ion accumulation < 0.05, signal to background >= 4, dark orange) results. Numbers of available CVs per boxplot are indicated. Center line, median; box limits, upper and lower quartiles; whiskers, 1.5x interquartile range. **j**, Heatmap representation of areas under the ROC (AUROC) for all (15) pairwise differential expression analyses using limma after missing value imputation by five commonly used imputation methods for MaxQuant data. Imputation by bpca resulted in best AUROC. **k**, Mean true positive rates for MaxQuant (grey, protein-level DE), DIA (red, peptide-level DE) and IceR (orange, peptide-level DE) outputs over true spike ratio of pairwise differential expression analyses. **l**, Mean false positive rates for MaxQuant (grey, protein-level DE), DIA (red, peptide-level DE) and IceR (orange, peptide-level DE) outputs over true spike ratio of pairwise differential expression analyses.

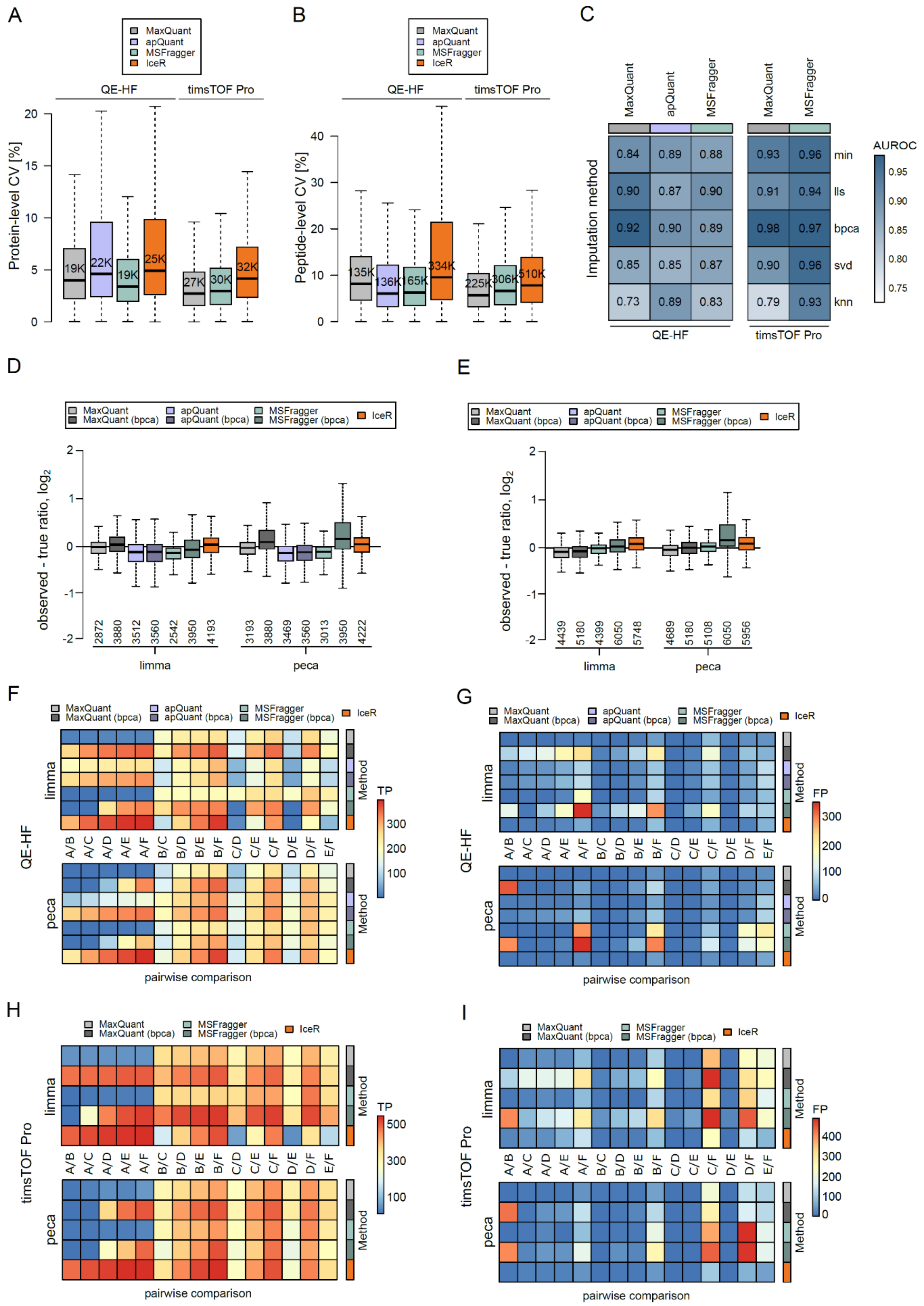

Supplementary Fig. 9 – Application of IceR to timsTOF Pro proteomics data.

a, Coefficient of variation of protein quantifications in MaxQuant (grey), apQuant (purple), MSFragger (green), and IceR (orange) results in QE-HF and timsTOF Pro data. Numbers of available CVs per

boxplot are indicated. Center line, median; box limits, upper and lower quartiles; whiskers, 1.5x interquartile range. **b**, as in a but showing CVs of peptide quantifications. **c**, Heatmap representation of areas under the ROC (AUROC) for all (15) pairwise differential expression analyses using limma after missing value imputation by five commonly used imputation methods for MaxQuant (grey), apQuant (purple), and MSFragger (green) in QE-HF and timsTOF Pro data. Imputation by bpca resulted in overall best AUROC. **d**, Deviation of determined from true protein abundance ratios of pairwise differential expression analyses on protein-level (limma) and peptide-level (peca) in MaxQuant (grey), MaxQuant with bpca imputation (dark grey), apQuant (purple), apQuant with bpca imputation (dark purple), MSFragger (green), MSFragger with bpca imputation (dark green), and IceR (orange) for QE-HF data. Numbers of spiked proteins for which abundance ratios could be determined per condition are indicated. Center line, median; box limits, upper and lower quartiles; whiskers, 1.5x interquartile range. **e**, as in d but showing results for timsTOF Pro data. **f**, Numbers of detected true positives on protein-level (limma) and peptide-level (peca) by respective quantification method for each individual pairwise comparison on QE-HF data. Spike-in amounts relative to human backround in %: A = 0, B = 3, C = 4.5, D = 6, E = 7.5, F = 9. **g**, As in f but showing numbers of detected false positives. **h**, As in f but showing numbers of detected true positives in case of timsTOF Pro data. **i**, As in f but showing numbers of detected false positives in case of timsTOF Pro data.

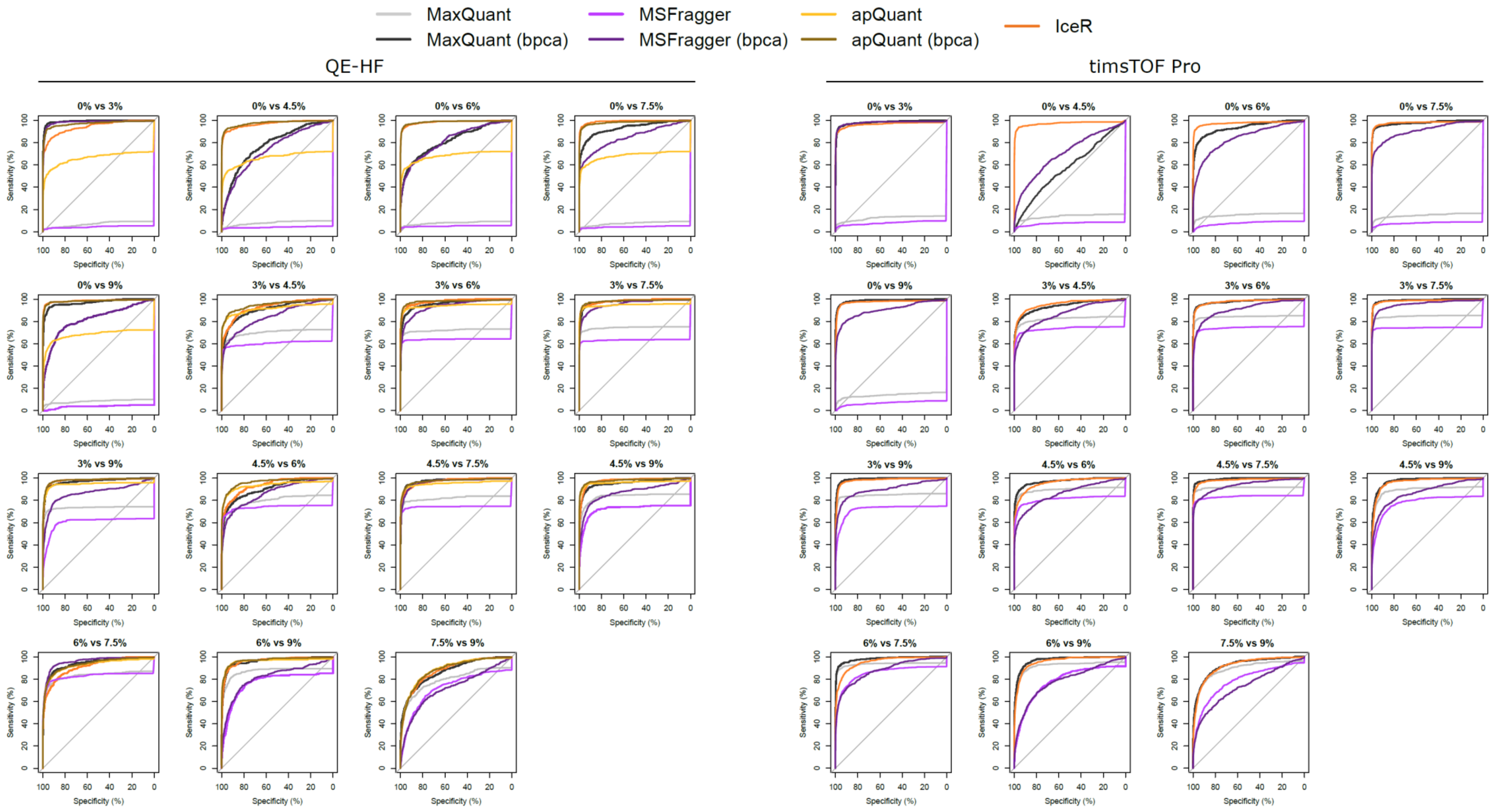

Supplementary Fig. 10 – All pairwise ROC curves for in-house QE-HF and timsTOF Pro data set.

All pairwise ROC curves on peptide-level differential testing for QE-HF and timsTOF Pro data for MaxQuant (grey), MaxQuant with bpca imputation (dark grey), apQuant (yellow), apQuant with bpca imputation (dark yellow), MSFragger (purple), MSFragger with bpca imputation (dark purple), and IceR (orange) data.

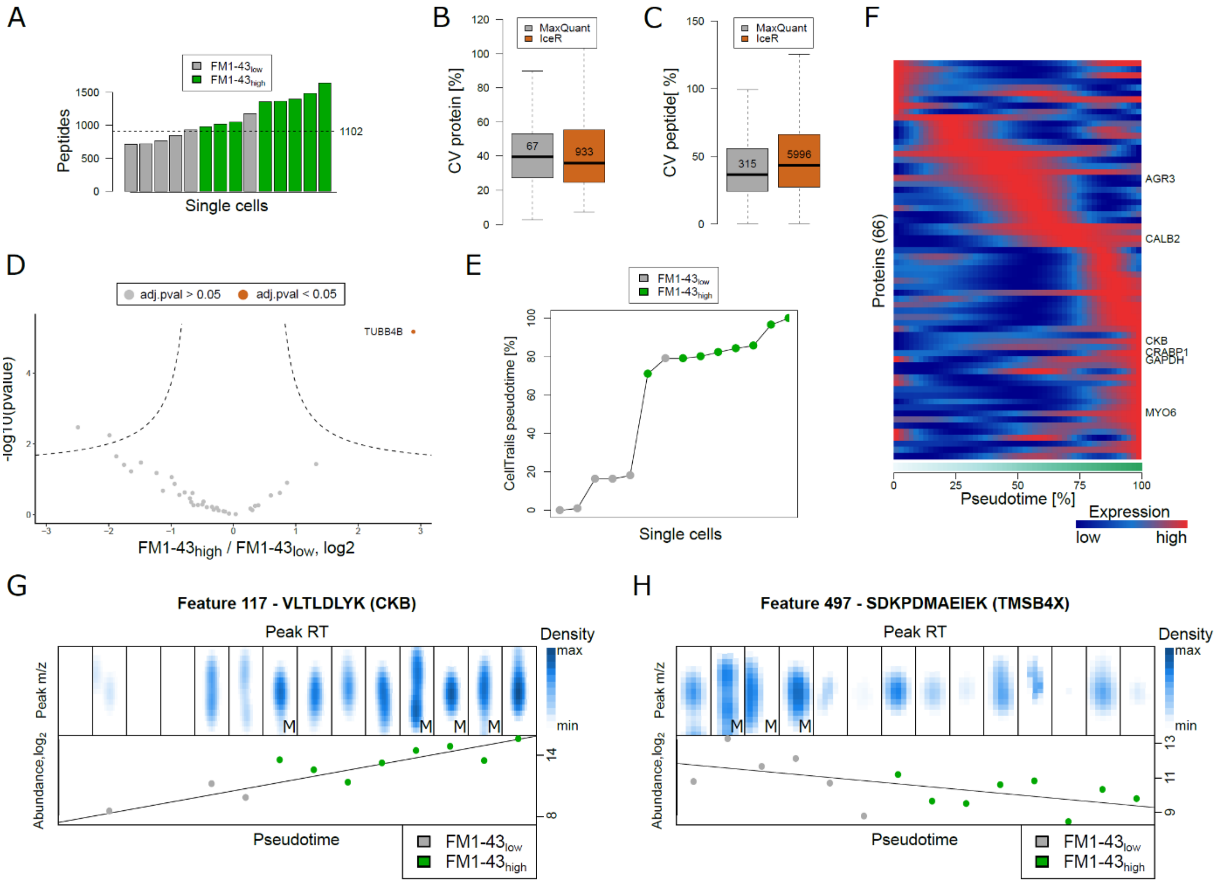

Supplementary Fig. 11 – Application of IceR to a single-cell proteomics data set.

a, Numbers of IceR features per single cell sample with significant (pvalue < 0.1) accumulation of ions. Dashed line indicates average over all samples. Single cell samples are coloured based on their FM1-43 uptake. **b**, Coefficient of variation of protein quantifications in MaxQuant (grey) and IceR (orange) data. Numbers of available CVs in respective data is indicated. Center line, median; box limits, upper and lower quartiles; whiskers, 1.5x interquartile range. **c**, Coefficient of variation of peptide quantifications in MaxQuant (grey) and IceR (orange) data. Numbers of available CVs in respective data is indicated. Center line, median; box limits, upper and lower quartiles; whiskers, 1.5x interquartile range. **d**, Volcano plot showing detected significantly (orange, adj. pvalue < 0.05) differently abundant proteins between single hair cells and single progenitor cells in MaxQuant data. Significance cut-off is indicated by a dashed line. **e**, Chronological ordering of single cells as a function of CellTrails' inferred pseudotime from MaxQuant data. Single cells are coloured according to their FM1-43 uptake. **f**, Scaled expression dynamics over pseudotime for all analyzed proteins in MaxQuant data based on generalized additive models (GAM). Low and high temporal protein expression is indicated by blue and red colour tones, respectively. **g**, Ion density in IceR-selected DICE windows per single-cell sample (upper panels) for a peptide (VLTLDLYK) of CKB (hair cell marker protein) and corresponding peptide abundances (lower panel) ordered by CellTrails` inferred pseudotime. Samples in which the peptide was identified by MaxQuant are indicated with an M in the corresponding upper panels. **h**, As in g but for a peptide (SDKPDMAEIEK) of TMSB4X (progenitor cell marker protein).

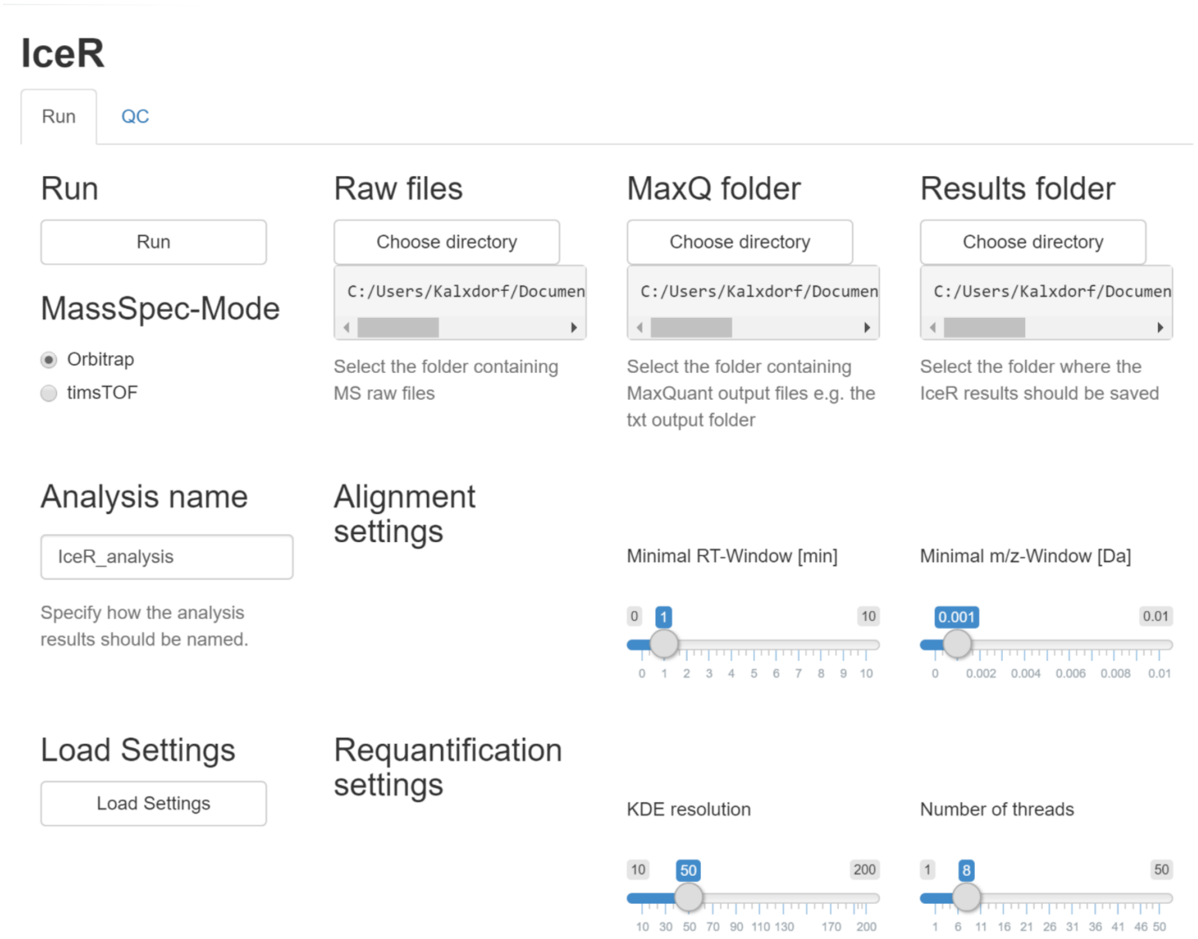

Supplementary Fig. 12 – Graphical user interface of IceR.

Graphical user interface of IceR allowing easy usage of IceR. The user can specify input (raw MS data, MaxQuant result data) and output (IceR result) folders, define which Mass spectrometer mode should be used (Orbitrap or timsTOF Pro), specify a run name, and adjust few run parameters like minimal RT-window size, minimal m/z-window size, kernel-density estimation resolution and number of threads.

Supplementary References

1. Choi, M. *et al.* ABRF Proteome Informatics Research Group (iPRG) 2015 Study: Detection of Differentially Abundant Proteins in Label-Free Quantitative LC-MS/MS Experiments. *J. Proteome Res.* **16**, 945–957 (2017).

2. Zhang, B., Käll, L. & Zubarev, R. A. DeMix-Q: Quantification-Centered Data Processing Workflow. *Mol. Cell. Proteomics MCP* **15**, 1467–1478 (2016).

3. Silva, J. C., Gorenstein, M. V., Li, G.-Z., Vissers, J. P. C. & Geromanos, S. J. Absolute quantification of proteins by LCMSE: a virtue of parallel MS acquisition. *Mol. Cell. Proteomics MCP* **5**, 144–156 (2006).

4. Cox, J. *et al.* Accurate proteome-wide label-free quantification by delayed normalization and maximal peptide ratio extraction, termed MaxLFQ. *Mol. Cell. Proteomics MCP* **13**, 2513–2526 (2014).

5. Suomi, T., Corthals, G. L., Nevalainen, O. S. & Elo, L. L. Using Peptide-Level Proteomics Data for Detecting Differentially Expressed Proteins. *J. Proteome Res.* **14**, 4564–4570 (2015).
