# Supplementary material for: IceR improves proteome coverage and data completeness in global and single-cell proteomics
Source: Nat Commun. 2021 Aug 9;12:4787. doi: 10.1038/s41467-021-25077-6 (PMC8352929; doi:10.1038/s41467-021-25077-6)
Supplement: Supplementary file 3 — Description of Additional Supplementary Files [file 41467_2021_25077_MOESM3_ESM.pdf]

Description of Additional Supplementary Files

Supplementary Data 1: Source data for Supplementary Fig. 2-3. Contains quantification results for the iPRG2015 study data set from DeMixQ, MaxQuant and IceR.

Supplementary Data 2: Source data for Fig. 1 and Supplementary Fig. 4. Contains quantification results for the data set from Ramus et al. from MaxQuant, MSFragger, apQuant, and IceR.

Supplementary Data 3: Source data for Fig. 2 and supplementary Fig. 4. Contains quantification results for the data set of Shen et al. from IonStar, MaxQuant, MSFragger, apQuant, and IceR.

Supplementary Data 4: Source data for Fig. 3-5 and Supplementary Fig. 7-10. Contains quantification results for the in-house generate E.coli spike-in data set from MaxQuant, MSFragger, apQuant, and IceR for QE-HF and timsToF Pro data.

Supplementary Data 5: Source data for Fig. 4 and Supplementary Fig. 8. Contains quantification results for the data sets from Bruderer et al. and the in-house DIA data set from MaxQuant and IceR.

Supplementary Data 6: Source data for Fig. 6. Contains quantification results for the Geyer et al. data set from MaxQuant and IceR.

Supplementary Data 7: Source data for Fig. 7 and Supplementary Fig. 11. Contains quantification results for the single-cell proteomics data set of Zhu et al. from MaxQuant and IceR.
