# Supplementary material for: IceR improves proteome coverage and data completeness in global and single-cell proteomics
Source: Nat Commun. 2021 Aug 9;12:4787. doi: 10.1038/s41467-021-25077-6 (PMC8352929; doi:10.1038/s41467-021-25077-6)
Supplement: Supplementary file 11 — Reporting Summary [file 41467_2021_25077_MOESM11_ESM.pdf]

Reporting Summary

Nature Portfolio wishes to improve the reproducibility of the work that we publish. This form provides structure for consistency and transparency in reporting. For further information on Nature Portfolio policies, see our [Editorial Policies](#) and the [Editorial Policy Checklist](#).

Statistics

For all statistical analyses, confirm that the following items are present in the figure legend, table legend, main text, or Methods section.

n/a Confirmed

- ☐ ☒ The exact sample size (n) for each experimental group/condition, given as a discrete number and unit of measurement
- ☐ ☒ A statement on whether measurements were taken from distinct samples or whether the same sample was measured repeatedly
- ☐ ☒ The statistical test(s) used AND whether they are one- or two-sided
Only common tests should be described solely by name; describe more complex techniques in the Methods section.
- ☒ ☐ A description of all covariates tested
- ☐ ☒ A description of any assumptions or corrections, such as tests of normality and adjustment for multiple comparisons
- ☐ ☒ A full description of the statistical parameters including central tendency (e.g. means) or other basic estimates (e.g. regression coefficient) AND variation (e.g. standard deviation) or associated estimates of uncertainty (e.g. confidence intervals)
- ☐ ☒ For null hypothesis testing, the test statistic (e.g. F , t , r) with confidence intervals, effect sizes, degrees of freedom and P value noted
Give P values as exact values whenever suitable.
- ☒ ☐ For Bayesian analysis, information on the choice of priors and Markov chain Monte Carlo settings
- ☒ ☐ For hierarchical and complex designs, identification of the appropriate level for tests and full reporting of outcomes
- ☒ ☐ Estimates of effect sizes (e.g. Cohen's d , Pearson's r), indicating how they were calculated

Our web collection on [statistics for biologists](#) contains articles on many of the points above.

Software and code

Policy information about [availability of computer code](#)

Data collection Q Exactive Orbitrap HF (Thermo Fischer Scientific) and timsTOF Pro (Bruker) were used for data acquisition.

Data analysis Raw DDA data was processed using MaxQuant (1.5.1.2 or 1.6.14), apQuant 3.1 with Proteome Discoverer 2.4, and MSFragger 3.1.1 with FragPipe 14.0 and raw DIA data was processed using Spectronaut (14.0). DDA data was further processed using Icer as described in the Materials and Methods section (V0.9.9, <https://github.com/mathiaskalxdorf/Icer/>). Data analysis was performed using R (V4.0.2). Missing value imputation was performed using the methods min, lls, bpca, svd, and knn (R-packages imputeLCMD V2.0 and pcaMethods V1.80.0). Differential expression analyses were performed using limma (R-package limma, V3.44.3) and PECA (R-package PECA, V1.22.0). Receiver operating characteristics analyses were performed using functions from pROC (R-package pROC, V1.16.2). Dimensional reduction for visualization of single cell data was performed using t-distributed stochastic neighbor embedding (tSNE, R-package tsne, version 0.1.3). Clustering of single cells was evaluated using the Silhouette score (R-package cluster, version 2.1.0). Unsupervised de novo chronological ordering of cells was performed using the R-package CellTrails (version 1.4.0).

For manuscripts utilizing custom algorithms or software that are central to the research but not yet described in published literature, software must be made available to editors and reviewers. We strongly encourage code deposition in a community repository (e.g. GitHub). See the Nature Portfolio [guidelines for submitting code & software](#) for further information.

Data

Policy information about [availability of data](#)

All manuscripts must include a [data availability statement](#). This statement should provide the following information, where applicable:

- Accession codes, unique identifiers, or web links for publicly available datasets
- A description of any restrictions on data availability
- For clinical datasets or third party data, please ensure that the statement adheres to our [policy](#)

Source data are provided with this paper. The mass spectrometry proteomics data generated for this study have been deposited to the ProteomeXchange Consortium via the PRIDE partner repository with the dataset identifier PXD019777 [<https://www.ebi.ac.uk/pride/archive/projects/PXD019777>] and PXD021425 [<https://www.ebi.ac.uk/pride/archive/projects/PXD021425>]. Protein sequences were taken from the UniProt database (<https://www.uniprot.org/>).

Raw LC-MS/MS data of the individual publicly available data sets were downloaded from respective sources:

1. iPRG2015 [ftp://iprg_study:ABRF329@ftp.peptideatlas.org/]
2. Ramus et al. PXD001819 [<http://proteomecentral.proteomexchange.org/cgi/GetDataset?ID=PX001819>]
3. Shen et al. PXD003881 [<http://proteomecentral.proteomexchange.org/cgi/GetDataset?ID=PX003881>]
4. Bruderer et al. [<ftp://PASS00589:WF6554orn@ftp.peptideatlas.org/>]
5. Zhu et al. PXD014256 [<http://proteomecentral.proteomexchange.org/cgi/GetDataset?ID=PX014256>]
6. Geyer et al. PXD002854 [<http://proteomecentral.proteomexchange.org/cgi/GetDataset?ID=PX002854>]

Published quantitative workflow results were downloaded from respective sources:

- DeMix-Q – iPRG2015 data set - <https://www.mcponline.org/lookup/suppl/doi:10.1074/mcp.O115.055475/-/DC1/mcp.O115.055475-1.xlsx>
- IonStar – Shen et al. data set - https://www.pnas.org/highwire/filestream/808767/field_highwire_adjunct_files/2/pnas.1800541115.sd02.xlsx
- HRM-DIA - Bruderer et al. data set - https://www.mcponline.org/highwire/filestream/35126/field_highwire_adjunct_files/2/mcp.M114.044305-3.xlsx

Field-specific reporting

Please select the one below that is the best fit for your research. If you are not sure, read the appropriate sections before making your selection.

- ☒ Life sciences ☐ Behavioural & social sciences ☐ Ecological, evolutionary & environmental sciences

For a reference copy of the document with all sections, see [nature.com/documents/nr-reporting-summary-flat.pdf](https://www.nature.com/documents/nr-reporting-summary-flat.pdf)

Life sciences study design

All studies must disclose on these points even when the disclosure is negative.

Sample size	Appropriate power for discovery of true positive events was gained from three or more biological replicates. Sample sizes were predefined due to the use of the publicly available data sets (e.g. Ramus et al. or Shen et al., see link to the data in the Data section above) or were chosen to be in line with these publicly available data sets.
Data exclusions	No data were excluded from the analyses.
Replication	Conclusions were drawn from reproducible effects in all replicates of the data sets. Respective numbers of available replicates are indicated in the manuscript, methods, and corresponding figure legends.
Randomization	This is not relevant to our study, as the study utilizes three or more biological replicates of lysates from cell lines with no covariates such as e.g. different patients.
Blinding	This is not relevant to our study, since data was analyzed using unbiased statistics and no covariates exist that could be analyzed with a certain bias.

Reporting for specific materials, systems and methods

We require information from authors about some types of materials, experimental systems and methods used in many studies. Here, indicate whether each material, system or method listed is relevant to your study. If you are not sure if a list item applies to your research, read the appropriate section before selecting a response.

Materials & experimental systems

n/a	Involvement in the study
☒	☐ Antibodies
☐	☒ Eukaryotic cell lines
☒	☐ Palaeontology and archaeology
☒	☐ Animals and other organisms
☒	☐ Human research participants
☒	☐ Clinical data
☒	☐ Dual use research of concern

Methods

n/a	Involvement in the study
☒	☐ ChIP-seq
☒	☐ Flow cytometry
☒	☐ MRI-based neuroimaging

Eukaryotic cell lines

Policy information about [cell lines](#)

Cell line source(s)	HeLa cells were from ATCC
Authentication	Cell line was not authenticated
Mycoplasma contamination	Cell line was not tested for mycoplasma contamination
Commonly misidentified lines<br>(See ICLAC register)	To the best of our knowledge no misidentified cell lines have been used in this study.
